# World Health Assembly 73: A Step Forward for Global Surgery

**DOI:** 10.5334/aogh.3237

**Published:** 2021-02-15

**Authors:** Gabrielle L. Cahill, Makela C. Stankey, Craig D. McClain, Kee B. Park

**Affiliations:** 1 Program in Global Surgery and Social Change, Harvard Medical School, Boston, MA, USA; 2 University of California, San Diego School of Medicine, La Jolla, CA; 3 Keck School of Medicine at the University of Southern California, Los Angeles, CA; 4 Department of Anesthesiology, Critical Care and Pain Medicine, Boston Children’s Hospital, Boston, MA

## Abstract

Member States at this year’s World Health Assembly 73 (WHA73), held virtually for the first time due to the COVID-19 pandemic, passed multiple resolutions that must be considered when framing efforts to strengthen surgical systems. Surgery has been a relatively neglected field in the global health landscape due to its nature as a cross-cutting treatment rather than focusing on a specific disease or demographic. However, in recent years, access to essential and emergency surgical, obstetric, and anesthesia care has gained increasing recognition as a vital aspect of global health. The WHA73 Resolutions concern specific conditions, as has been characteristic of global health practice, yet proper care for each highlighted disease is inextricably linked to surgical care. Global surgery advocates must recognize how surgical system strengthening aligns with these strategic priorities in order to ensure that surgical care continues to be integrated into efforts to decrease global health disparities.

The importance of surgical, obstetric, and anesthesia (SOA) care has become increasingly recognized within the global health landscape, with 2015 marking a pivotal year for global surgery. That year, the World Bank’s third edition of Disease Control Priorities (DCP3) included its first volume on Essential Surgery, the Lancet Commission on Global Surgery released its report, and the United Nations set its Sustainable Development Goals (SDGs), with four major targets of SDG3 unachievable without increasing access to surgery [1]. In the same year, the 68th World Health Assembly (WHA68) unanimously passed WHA Resolution 68.15, signifying a global commitment to strengthen surgical and anesthesia care as an essential part of universal health coverage (UHC) [2].

Since 2015, several important international resolutions and decisions at the subsequent World Health Assemblies have further integrated surgical and anesthesia care as an essential part of resilient health systems. In 2017, WHA Decision 70(22) was passed, affirming the need to collect robust data on emergency and essential surgical and anesthesia care and requiring Member States, the governing body of the World Health Assembly, to report back to the WHA every two years on their progress [3]. This need for a comprehensive approach to scaling up of surgical and anesthesia services at the national level has resulted in the rapid adoption of the National Surgical, Obstetric, and Anesthesia Plan (NSOAP) process, culminating in a strategic plan that is meant to be incorporated into national health plans. Since 2017 several low- and middle-income countries (LMICs) have adopted NSOAPs as a means to further develop surgical care [1].

The 73rd World Health Assembly this year was unique due to the COVID-19 pandemic. The Member States have had to shift their agendas to deal with a global pandemic and accommodate a new, socially distanced normal while continuing their quest to improve health around the world. The WHA 73 agenda passed five resolutions addressing the following areas: COVID-19, cervical cancer, eye health, tuberculosis, and food security. Of the five resolutions, three (COVID-19, cervical cancer, and eye health) necessitate the continued development and integration of surgical and anesthesia care within health systems. Here we explore this year’s resolutions and discuss their implications for global surgery’s continued growth.

Unsurprisingly, the first resolution (Resolution 73.1) was about COVID-19, calling for “intensification of cooperation and collaboration at all levels” to contain the pandemic and mitigate its impact [4]. The pandemic has necessitated a rapid and massive scale-up of critical care capacity—by four to five times in some countries [5]. Many elements required for critical care are integral aspects of functional surgical systems, such as reliable power, anesthesia capacity, and intensive care units. Though building surgical capacity requires more investment in highly trained personnel and infrastructure than some other global health interventions, surgical scale-up has been shown to be cost-effective [6]. The pandemic has highlighted that strong surgical systems positively impact health systems’ ability to react quickly and effectively in times of surging healthcare needs. As governments grapple with system failures and work to build COVID-19 response capacity as well as future pandemic preparedness, this is key to highlight. Investment in surgical expansion not only reduces the disparities, morbidity, and mortality from surgically treatable disease, but also aids nations in their efforts to build pandemic response capacity.

For the first time ever, the Member States of the WHA have set the eradication of a cancer as a priority—Resolution 73.2 recognizes the urgent need to “accelerate the elimination of cervical cancer,” including surgical treatment for invasive disease [7]. From a strategic standpoint, as countries continue developing their surgical systems around the world, this new emphasis on the vaccination, detection, and treatment of cervical cancer represents areas of shared interest and potential cooperation between the two communities of practice. For example, NSOAPs should clearly incorporate the workforce, infrastructure, informational management, financing, governance, and service delivery aspects of cervical cancer treatment that need to be in place to treat the surge of anticipated cervical cancer cases from increased detection efforts. Increased funding toward the elimination of cervical cancer should, at least in part, be directed toward surgical system strengthening. The global surgery community could work hand in hand with the cervical cancer community to co-advocate for each other’s causes.

Finally, building on six previous WHA resolutions on eye care, Resolution 73.4 notes the need to address preventable vision impairment and blindness, and specifically mentions effective coverage of cataract surgery in its requests of the Director-General [8]. Global surgery has not routinely included eye care among its priorities, possibly because cataract surgery may not require the same developments in infrastructure as some other surgical interventions. However, it remains that cataracts are a surgical condition causing major morbidity, especially in LMICs, which require a specialized, highly trained workforce for effective care and share much of the resources used in essential and emergency surgical care. As countries develop comprehensive strategic plans to enhance surgical care, local stakeholders should ensure eye surgeons and eye care champions are intentionally included as part of policy development and implementation.

The WHA 73 resolutions reflect the need for the continued development of surgical systems as part of robust health systems in achieving universal health coverage. There is a clear political will to develop policies to improve equity and availability of treatment for these diseases. Global surgery advocates must recognize how surgical strengthening aligns with these resolutions, where global surgery goals synergize with the WHA and broader global health priorities, and seek to partner with these stakeholders to ensure continued integration of surgical care into efforts to reduce health disparities globally.
